# Unraveling the Causal Association Between Circulating Copper Levels and Erectile Dysfunction: A Comprehensive Analysis via Bidirectional Two Sample Mendelian Randomization Study

**DOI:** 10.1002/fsn3.70247

**Published:** 2025-05-26

**Authors:** Zilong Wang, Zhen Xu, Meilu Li, Zhenghao Li, Dandan Li, Changze Song, Xiaobin Wang

**Affiliations:** ^1^ Department of Burns and Plastic Surgery The Seventh Affiliated Hospital, Sun Yat‐sen University Shenzhen China; ^2^ Scientific Research Center The Seventh Affiliated Hospital, Sun Yat‐sen University Shenzhen China; ^3^ Department of Andrology The Seventh Affiliated Hospital, Sun Yat‐sen University Shenzhen China; ^4^ Institute of Molecular Physiology Shenzhen Bay Laboratory Shenzhen China; ^5^ Division of Rheumatology, Department of Medicine Mayo Clinic Rochester Minnesota USA; ^6^ Department of Dermatology The Seventh Affiliated Hospital, Sun Yat‐sen University Shenzhen China; ^7^ Department of Dermatology The Second Hospital of Harbin Medical University, Harbin Medical University Harbin China; ^8^ Department of Radiation Therapy Dongguan Hospital of Guangzhou University of Chinese Medicine Dongguan China; ^9^ Department of Urology The Affiliated Hospital, School of Medicine, Southern University of Science and Technology Shenzhen China

**Keywords:** circulating copper, erectile dysfunction, mendelian randomization study, micronutrition, nutritional balance

## Abstract

Erectile dysfunction (ED) is a prevalent condition severely impacting men's quality of life. While micronutrient balance is critical for sexual health, observational studies on micronutrient and ED remain inconclusive. The objective of this study was to evaluate the causal role of circulating levels of copper and other micronutrients in ED risk by Mendelian randomization (MR). We conducted bidirectional two‐sample MR using summary statistics from European‐ancestry cohorts involving thirteen micronutrients. Inverse‐variance weighted (IVW) MR was complemented by sensitivity analyses, including MR‐Egger and MR‐PRESSO, and pooled analysis of two GWAS‐based datasets was performed to substantiate the findings of MR. In this study, circulating copper levels significantly increased ED risk in the discovery (OR = 1.129, 95% CI: 1.004–1.27, *p* = 0.042) and replication (OR = 1.115, 95% CI: 1.001–1.242, *p* = 0.0476) stages, as well as pooled analyses (OR = 1.122, 95% CI: 1.036–1.214, *p* = 0.0046). Sensitivity analyses reinforced the robustness of these findings, with no significant heterogeneity or directional pleiotropy observed. Reverse MR found no causal effect of ED on micronutrients. In summary, this study provides robust genetic evidence that elevated circulating copper levels are a modifiable risk factor for ED. Personalized management of copper intake, guided by genetic predisposition, may mitigate ED risk and improve sexual health outcomes. These findings highlight the clinical relevance of copper homeostasis in ED prevention and underscore the need for targeted nutritional interventions.

## Graphical abstract

Erectile dysfunction (ED) is a common and debilitating condition that negatively impacts men's quality of life and psychological well‐being. Excessive circulating copper levels are causally linked to an increased risk of ED, as shown by Mendelian randomization and meta‐analysis. Balanced copper intake is crucial for sexual health, highlighting the need for careful nutritional management to prevent ED risks.

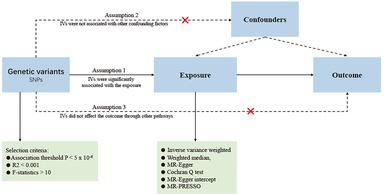

**Abbreviations**
cGMP: cyclic guanosine monophosphate
CI: confidence interval
CP: ceruloplasmin
ED: erectile dysfunction
eNOS: endothelial nitric oxide synthase
GC: guanylate cyclase
GTP: guanosine triphosphate
GWAS: genome‐wide association studies
IV: instrumental variables
IVW: inverse‐variance weighted
MEC‐IEU: Medical Research Council‐Integrative Epidemiology Unit
MR: Mendelian randomization
NO: nitric oxide
ONOO: peroxynitrite
OR: odds ratios
PDE5i: phosphodiesterase‐5 inhibitors
ROS: reactive oxygen species
SNP: single nucleotide polymorphisms
SOD: superoxide dismutase

## 1 Introduction

Erectile dysfunction (ED) is a complex and multifactorial andrological disorder, defined by the persistent inability to achieve or sustain a satisfactory erection (Minhas et al. 2021). This condition profoundly influences both the physical and psychological well‐being of patients, while also adversely affecting the stability of intimate partner relationships and the overall cohesion of family life (Minhas et al. 2021). As the global population ages and lifestyle patterns continue to evolve, the prevalence of ED is projected to increase substantially, with an estimated 322 million cases anticipated worldwide by 2025 (Ayta et al. 1999). In Asia, it is projected that over 113 million men will be affected by ED, which will lead to a diminished quality of life for many and a substantial escalation in healthcare expenditures, thereby presenting considerable socio‐economic challenges for the region. Despite the increasing prevalence of ED, the limited comprehension of its precise etiology and associated risk factors has significantly constrained the effectiveness of current treatment modalities. Unfortunately, the precise etiologies and risk factors of ED have not been elucidated. Phosphodiesterase‐5 inhibitors (PDE5i), which are the first‐line pharmacological treatment for ED (Minhas et al. 2021), demonstrate limited efficacy, with patient satisfaction rates falling below 50% (Moncada et al. 2018). Moreover, alternative therapeutic approaches, including surgical implants and stem cell transplants, are associated with risks such as infection and mechanical failure, which hinder their widespread adoption (Xuan et al. 2007).

Recent research indicates that micronutrients, encompassing essential minerals and vitamins, are modifiable risk factors for ED and are pivotal in its prevention and treatment (Chen et al. 2019). Some observational studies have identified correlations between deficiencies in micronutrients, such as magnesium, zinc, selenium (Liu et al. 2022), folic acid, and vitamin B12 (Chen et al. 2019; Xu et al. 2021), and the onset of ED. Nevertheless, the conclusions drawn from these studies are largely suggestive and constrained by several limitations. Primarily, observational studies are frequently affected by confounding variables, including age, lifestyle, and common comorbidities such as diabetes and cardiovascular diseases. These variables may concurrently influence both micronutrient levels and the occurrence of ED, thereby complicating the determination of a definitive causal relationship between micronutrients and ED. Furthermore, observational studies are susceptible to reverse causality, rendering it ambiguous whether micronutrient deficiencies contribute to ED or if ED, arising from other health conditions, influences micronutrient levels. Additionally, these studies are constrained in their capacity to control for long‐term nutritional exposure and to thoroughly account for all potential confounding variables, thereby limiting the reliability of causal inferences. Although these associative findings are compelling, more rigorous research methodologies are necessary to ascertain whether micronutrients exert a direct effect on ED.

Mendelian randomization (MR) represents an innovative methodological approach that employs genetic variants as instrumental variables (IVs) to evaluate causal relationships between exposures and outcomes, thereby reducing the influence of confounding factors (Sheehan et al. 2008). By utilizing data from genome‐wide association studies (GWAS), MR facilitates the robust estimation of the causal effects of micronutrients on clinical outcomes, effectively addressing the limitations inherent in traditional observational studies (Emdin et al. 2017).

In this study, we employ a bidirectional two‐sample MR analysis to investigate potential causal relationships between circulating micronutrient levels and ED in both directions. Notably, genetic variants associated with circulating micronutrient levels act as proxies for lifelong exposure, analogous to sustained environmental intake (Burgess et al. 2013; Gill et al. 2019; Smith and Ebrahim 2003). These variants influence copper homeostasis independently of confounding factors, enabling causal inference consistent with MR's core assumptions (Hemani et al. 2018). Furthermore, we substantiate these findings through pooled analysis of two GWAS‐based datasets and sensitivity assessments, with the overarching objective of determining whether copper might serve as a viable target for clinical intervention and nutritional management of ED.

## 2 Materials and Methods

### 2.1 Study Design

**FIGURE 1:**
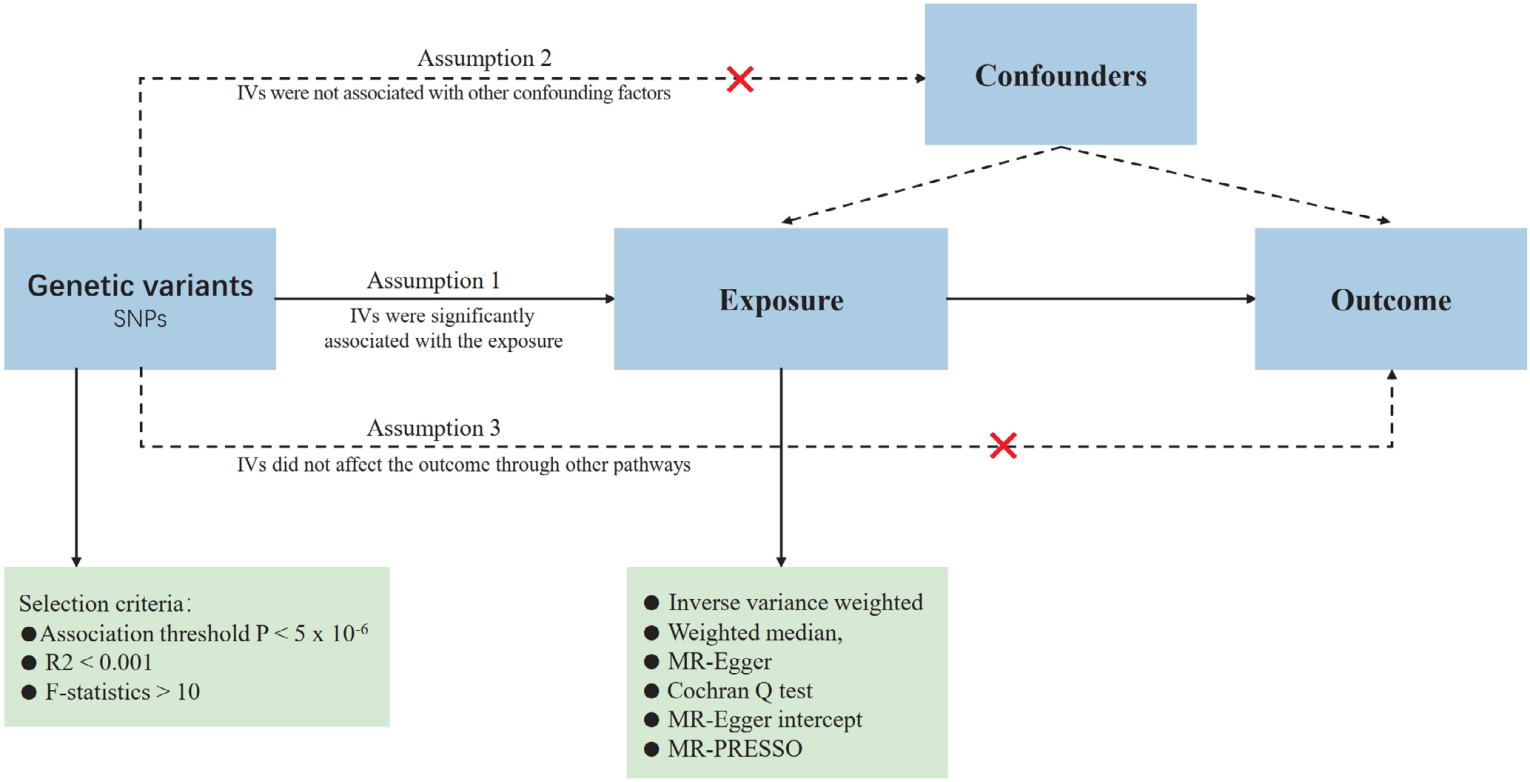
The flow chart of the study design for this MR study.

The MR study utilized summary‐level data obtained from genome‐wide association analyses focusing on circulating micronutrient levels and ED, as documented in published GWASs. A two‐sample MR analysis was conducted to investigate the impact of genetically predicted higher levels of micronutrients on the risk of ED. The analyzed micronutrients included calcium, copper, iron, magnesium, selenium, zinc, carotene, folate, vitamin B6, vitamin B12, vitamin C, vitamin D, and vitamin E. ED data were sourced from two independent large GWAS datasets, and a pooled analysis of two GWAS‐based datasets was performed on the combined data. Additionally, a reverse MR analysis was undertaken to investigate the association between genetic predisposition to ED and circulating micronutrient levels. To minimize potential bias from population stratification, both the micronutrient and ED cohorts were restricted to individuals of European ancestry. Importantly, there was no overlap between the cohorts focusing on micronutrient and ED. It is noteworthy that all data utilized in this study are publicly available and were obtained from studies with appropriate participant consent, thereby obviating the need for ethical approval for the current investigation. The flow chart of the study design for the MR study was illustrated in Figure 1.

### 2.2 Data on the Genetically Predicted Levels of Circulating Micronutrients

We conducted a comprehensive search for published GWASs assessing individuals of European ancestry in both the GWAS Catalog and PubMed (last accessed on December 23, 2023). Notably, no GWASs were identified for vitamins B1, B2, B3, B5, B7, sulfur, iodine, chloride, and fluoride. Furthermore, GWASs investigating vitamin K, potassium, sodium, cobalt, chromium, and molybdenum were excluded due to the absence of significant genome‐wide results. Four micronutrients of potential interest were identified: calcium, copper, selenium, and zinc. Data on iron, magnesium, carotene, folate, vitamin B6, vitamin B12, vitamin C, vitamin D, and vitamin E were sourced from the Medical Research Council‐Integrative Epidemiology Unit (MRC‐IEU) consortium, utilizing the following datasets: ukb‐b‐20,447, ukb‐b‐7372, ukb‐b‐16,202, ukb‐b‐11,349, ukb‐b‐7864, ukb‐b‐19,524, ukb‐b‐19,390, ukb‐b‐18,593, ukb‐b‐6888, respectively. Notably, the copper dataset utilized erythrocyte‐derived copper measurements. Erythrocyte copper levels reflect long‐term exposure and homeostasis, as red blood cells accumulate metals over their lifespan (120 days), offering distinct advantages over serum or plasma measurements for assessing chronic effects. Technical details on metal quantification in erythrocytes, including sample preparation and analytical validation, are described by Heitland and Köster (Heitland and Koster 2021). All GWAS summary statistics utilized in this work are publicly available through the IEU Open GWAS database (https://gwas.mrcieu.ac.uk/). Specific dataset identifiers are comprehensively documented in Table 1.

**TABLE 1:** Summary statistics of the micronutrients and erectile dysfunction GWAS dataset.

Phenotype	Sources	Sample size	Number of variants	Consortium/first author	Year	Domain
Erectile dysfunction (discovery stage)	—	95,178	16,378,833	FINN	2021	European
Erectile dysfunction (replication stage)	—	223,805	9,310,196	Bovijn J	2018	European
Calcium	Serum	400,792	4,218,949	Barton AR	2021	European
Copper	Erythrocyte	2603	2,543,646	Evans	2013	European
Iron	Serum	64,979	9,851,867	MRC‐IEU	2018	European
Magnesium	Serum	64,979	9,851,867	MRC‐IEU	2018	European
Selenium	Erythrocyte	2874	2,451,527	Evans	2013	European
Zinc	Erythrocyte	2603	2,543,610	Evans	2013	European
Carotene	Serum	64,979	9,851,867	MRC‐IEU	2018	European
Folate	Serum	64,979	9,851,867	MRC‐IEU	2018	European
Vitamin A	Serum	460,351	9,851,867	MRC‐IEU	2018	European
Vitamin B6	Plasma	64,979	9,851,867	MRC‐IEU	2018	European
Vitamin B12	Serum	64,979	9,851,867	MRC‐IEU	2018	European
Vitamin C	Plasma	64,979	9,851,867	MRC‐IEU	2018	European
Vitamin D	Serum	64,979	9,851,867	MRC‐IEU	2018	European
Vitamin E	Serum	64,979	9,851,867	MRC‐IEU	2018	European

Abbreviations: FINN, FinnGen Biobank; MRC‐IEU, Medical Research Council‐Integrative Epidemiology Unit.

### 2.3 Data on the Genetically Predicted Risk of ED

We have found publicly available summary statistics from two independent cohorts of European ancestry: FinnGen Biobank (dataset: finn‐b‐ERECTILE_DYSFUNCTION) (Kurki et al. 2023) as the discovery stage and GWAS conducted by Bovijn J et al. (dataset: ebi‐a‐GCST006956) as the replication stage (Bovijn et al. 2019). The sample sizes in the two stages are as follows: discovery stage (ED vs. non‐ED, 1154 vs. 94,024), replication stage (ED vs. non‐ED, 6175 vs. 217,630). In the Bovijn J dataset, ED was defined through three criteria: (1) ICD‐10 codes N48.4/F52.2 for clinical diagnosis, (2) self‐reported ED or use of phosphodiesterase‐5 inhibitors (sildenafil, tadalafil, vardenafil), and (3) surgical records of ED interventions (OPCS‐4 codes L97.1/N32.6). The study population included men from the UK Biobank (median age 59 years, 1.53% ED prevalence), Estonian Genome Center (42 years, 7.04% ED), and Partners HealthCare Biobank (65 years, 25.35% ED), with principal components analysis confirming European ancestry. Additionally, we performed a reverse‐MR analysis, considering ED as the exposure.

### 2.4 Instrumental Variable Selection

The selection of IV in MR analysis was based on the following 3 fundamental assumptions: (1) IVs that directly influence the exposure; (2) IVs have no associations with confounders; and (3) apart from exposure factors, IVs had no influence on outcomes through alternative pathways.

To investigate the above assumptions, the following conditions were met during single nucleotide polymorphism (SNP) screening: First, in order to ensure an adequate number of instrument SNPs (number > 3) shared between exposures and outcomes, we selected SNPs with genome‐wide suggestive significance (*p* < 5 × 10^−6^) from published GWAS databases that independently associated with circulating micronutrient concentrations. Second, independent variants were identified using a clumping procedure in R software, setting a linkage‐disequilibrium (LD) threshold of *r* ^2^ < 0.001 within a 10,000 kb distance to prevent LD among the remaining IVs for micronutrients. Third, we searched the ED‐related dataset for the SNPs corresponding to the micronutrients, and the noncorresponding or palindromic SNPs were deleted. Fourth, the strength of the IVs was assessed based on the *F* statistic, calculated using the formula: *F* = ((*N* − 2) × R2)/(1 − R2), where R2 represents the proportion of variance explained by the genetic instrument, and N is the sample size. The R2 value was computed using the formula: 2 × beta2 × EAF × (1 − EAF), where beta represents the effect estimate of the genetic variant in the exposure, measured in standard deviation (SD) units, and EAF represents the effect allele frequency. *F* value > 10 indicates the absence of weak IV bias, further supporting the correlation hypothesis. Following harmonization of SNPs by effect allele, we screened all instrument SNPs in the PhenoScanner database (http://www.phenoscanner.medschl.cam.ac.uk/) to assess potential confounding traits or horizontal pleiotropy in the exposure‐outcome association. Specifically, IVs for copper were rigorously validated for independence from zinc and other micronutrients. No SNPs associated with copper exhibited pleiotropic effects on zinc (all *p* > 0.05), confirming the specificity of the observed causal relationship and minimizing bias from shared genetic pathways.

For the reverse MR analysis, the criteria for SNP selection were consistent with those employed in the forward‐direction analysis.

### 2.5 Mendelian Randomization Analysis

For MR analysis, the IVW model was considered as the main method for obtaining results, which calculated estimates obtained from average multi‐SNP Wald ratios (SNP outcome association/SNP exposure association). However, recognizing that the IVW model yields the most precise estimates only under the assumption that all SNPs are valid instruments and any pleiotropy is balanced, we employed additional methods, including weighted median, MR‐Egger, weighted mode, and simple mode, to validate the results.

Furthermore, several sensitivity analyses were conducted to assess the robustness of the associations. The weighted median analysis was employed, providing a consistent, valid estimate if at least 50% of the IVs are valid. The MR‐Egger method, rooted in Egger regression, was utilized to detect directional pleiotropy by introducing an intercept term (*p* < 0.05 for intercept indicates pleiotropy) into the regression model. This method generates valid estimates of causal effects even when all instruments are invalid. MR pleiotropy residual sum and outlier (MR‐PRESSO) analysis was conducted to identify horizontal pleiotropic outliers and provide adjusted estimates. A *p*‐value below 0.05 for the Global test in MR‐PRESSO indicates the presence of horizontal pleiotropic outliers. Leave‐one‐out sensitivity analyses were employed to assess the impact of individual SNPs on causality estimates. Heterogeneity tests for MR‐Egger regression and the IVW method were performed using Cochran's *Q* statistics. *p* values > 0.05 are considered indicative of no significant heterogeneity or pleiotropy among the SNPs. The results are presented as odds ratios (ORs) and 95% confidence intervals (CIs).

### 2.6 Pooled Analysis of Two GWAS‐Based Datasets

Exposure‐specific MR analyses were independently performed within the outcome datasets of FinnGen Biobank (Kurki et al. 2023) and Bovijn et al. (2019), with subsequent pooled analysis of two GWAS‐based datasets combining estimates for each exposure's impact on ED risk. Both datasets represent retrospective, genetically informed analyses derived from published GWAS data sources. The choice of the effect model was based on the heterogeneity of results. In cases where there was little significant heterogeneity with *I* ^2^ ≤ 50%, the fixed‐effects model was applied to combine the results. For *I* ^2^ > 50%, there was great heterogeneity, and we used the random‐effects model to combine the results. The combined estimates from this pooled analysis were considered our primary evidence for causal inference. However, if only one reliable MR result was available, the final causality was based on it.

### 2.7 Statistical Analysis

To account for multiple testing, we employed a Bonferroni‐corrected significance threshold of 0.0038 (calculated as 0.05 divided by 13, representing the 13 types of micronutrients). *p* values falling between 0.0038 and 0.05 were deemed to indicate nominally causal associations between the exposures and the outcomes. All analyses were two‐sided and performed using the R language (version 4.2.0) with the “TwoSampleMR,” “MRPRESSO,” and “meta” packages.

## 3 Results

### 3.1 Circulating Copper Levels Are the Risk Factors of ED

We incorporated 470 SNPs that demonstrated a significant association with circulating minerals and vitamins (*p* < 5 × 10^−8^) and subsequently filtered them by removing those in linkage disequilibrium (*r* ^2^ < 0.001, 10,000 kb). These filtered SNPs were utilized as IVs in the MR analysis, with the GWAS datasets of FinnGen Biobank and Bovijn et al. serving as the discovery and replication stages, respectively.

**FIGURE 2:**
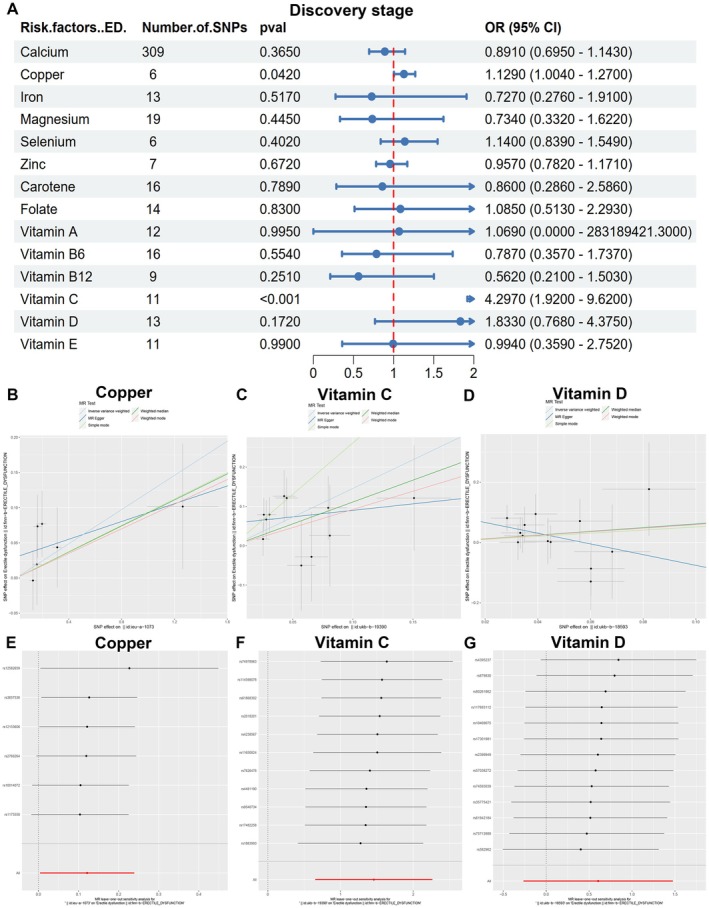
The causal association between micronutrition and erectile dysfunction in the discovery stage. (A) The forest diagram of MR study in the discovery stage. (B–D) Mendelian randomization study of the effects of copper (B), vitamin C (C) and vitamin D (D) on erectile dysfunction in the discovery stage. (E–G) Leave‐one‐out sensitivity analysis for this Mendelian randomization study of the effects of copper (E), vitamin C (F), and vitamin D (G) on erectile dysfunction in the discovery stage.

Utilizing the IVW method, we identified copper (OR = 1.129, 95% CI 1.004–1.27, *p* = 0.042) and vitamin C (OR = 4.297, 95% CI 1.92–9.62, *p* < 0.001) as potential causal factors associated with ED in the discovery stage (Figure 2A–C, Table S1). Conversely, circulating vitamin D demonstrated a weak correlation with ED (OR = 1.833, 95% CI 0.768–4.375, *p* = 0.172, Figure 2A,D). Importantly, no evidence of horizontal pleiotropy was detected by weighted median, MR‐Egger, MR‐Egger regression, and MR‐PRESSO methods (Figure S1, Table S1). The IVs employed in this stage of MR for circulating vitamin C, vitamin D, copper, and other micronutrients are detailed in Table 2 and Table S2. A leave‐one‐out sensitivity analysis demonstrated that the deletion of any single SNP related to copper, vitamin C, and vitamin D did not significantly alter the outcomes (Table 3, Figure 2E–G, Figure S2), thereby confirming the robustness of the findings. Cochran's *Q* test was applied to assess the heterogeneity among the selected SNPs, and the results showed that neither MR Egger nor IVW analysis had statistically significant heterogeneity (*p* > 0.05), except for carotene (Table S1).

**TABLE 2:** The instrumental variables for different circulating vitamin D and copper used in the MR analysis in the discovery and replication stage.

Exposure	SNP	CHR	Effect allele	Reference allele	Discovery stage of ED				Replication stage of ED			
					Beta	SE	*p*	*F*‐statistics	Beta	SE	*p*	*F*‐statistics
Vitamin D	rs10469075	18	T	C	−0.0009	0.0584	0.9873	21.42587224	−0.0382	0.0248	0.1235	21.42587224
Vitamin D	rs117693112	7	A	G	−0.0306	0.1566	0.8449	22.21523881	0.0371	0.0556	0.504001	22.21523881
Vitamin D	rs17301981	9	C	T	−0.0046	0.0761	0.9513	20.86827669	−0.038	0.0332	0.2518	20.86827669
Vitamin D	rs2399949	3	C	T	−0.0223	0.0566	0.692901	23.52129374	0.0055	0.0278	0.843	23.52129374
Vitamin D	rs35775421	14	A	G	−0.071	0.0729	0.3301	20.98288568	0.0034	0.0407	0.9341	20.98288568
Vitamin D	rs4395237	2	G	T	0.1294	0.0949	0.1728	22.18160959	0.0201	0.0535	0.707801	22.18160959
Vitamin D	rs57038272	5	T	C	0.0319	0.0523	0.5425	21.21319344	0.0099	0.0251	0.6947	21.21319344
Vitamin D	rs582962	6	A	G	−0.0809	0.0437	0.0643695	21.68655231	−0.0208	0.0214	0.3316	21.68655231
Vitamin D	rs61942184	12	C	G	0.1768	0.1548	0.2532	21.43681432	0.0779	0.0658	0.2362	21.43681432
Vitamin D	rs679830	7	C	T	0.0864	0.0913	0.3437	22.26165747	−0.0336	0.0451	0.4559	22.26165747
Vitamin D	rs74593039	10	C	G	0.0582	0.0601	0.333	21.55732844	0.0175	0.0274	0.5219	21.55732844
Vitamin D	rs75713989	3	T	C	0.0944	0.0664	0.155	22.97645139	0.028	0.0296	0.344	22.97645139
Vitamin D	rs80261862	7	T	C	−0.0015	0.0552	0.9787	23.44727144	0.0043	0.0319	0.894	23.44727144
Vitamin D	rs9328367	6	T	A	−0.0494	0.0429	0.2502	21.72516139	−0.0418	0.0201	0.0377103	21.72516139
Copper	rs10014072	4	G	A	−0.0735	0.0447	0.1003	23.24855935	−0.0505	0.0212	0.0174598	23.24855935
Copper	rs1175550	1	G	A	0.077	0.0474	0.1044	38.25574007	0.0388	0.0228	0.0882999	38.25574007
Copper	rs12153606	5	T	G	−0.0194	0.0577	0.7363	21.85257395	0.0323	0.0246	0.1907	21.85257395
Copper	rs12582659	12	C	T	0.1017	0.0892	0.2543	21.83018246	0.0525	0.1121	0.639699	21.83018246
Copper	rs2769264	1	G	T	0.0436	0.0569	0.4437	84.68315405	0.0393	0.025	0.1154	84.68315405
Copper	rs3857536	6	T	C	0.0035	0.0424	0.9352	21.20945661	−0.0155	0.0194	0.4241	21.20945661

Abbreviations: ED, erectile dysfunction; MR, Mendelian Randomization.

**TABLE 3:** The sensitivity analysis results regarding the association between micronutrients and ED.

Exposure	Outcome	Heterogeneity test				MR‐Egger		MR‐PRESSO	
		IVW Cochran's *Q*	*p* IVW	MR. Egger Cochran's *Q*	*p* MR. Egger	Intercept value	*p*	RSSobs	*p*
Discovery stage									
Vitamin D	Erectile dysfunction	10.100	0.607	7.769	0.734	0.102	0.155	11.907	0.621
Copper	Erectile dysfunction	3.233	0.664	2.192	0.701	0.0298	0.365	7.524	0.615
Erectile dysfunction	Vitamin D	10.484	0.487	10.423	0.404	−0.0011	0.813	12.094	0.548
Erectile dysfunction	Copper	6.129	0.190	5.049	0.168	−0.2456	0.482	9.928	0.226
Replication stage									
Vitamin D	Erectile dysfunction	4.364	0.976	4.247	0.962	0.011	0.739	5.110	0.972
Copper	Erectile dysfunction	7.633	0.178	7.304	0.121	0.0113	0.693	9.798	0.300
Erectile dysfunction	Vitamin D	11.607	0.170	11.578	0.115	−0.0013	0.897	16.121	0.139
Erectile dysfunction	Copper	/	/	/	/	/	/	/	/

Abbreviations: ED, erectile dysfunction; IVW, inverse variance‐weight; MR, Mendelian randomization.

**FIGURE 3:**
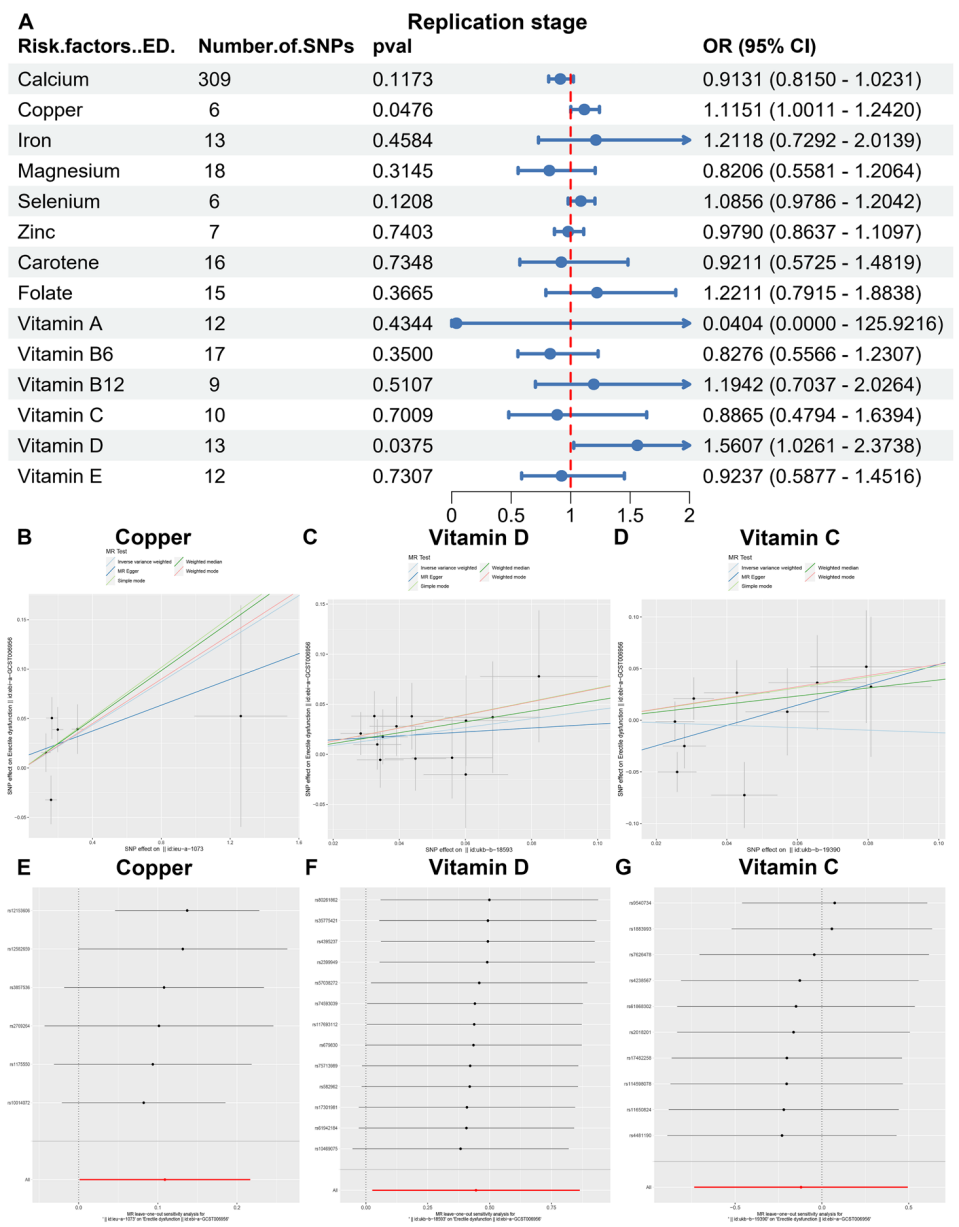
The causal association between micronutrition and erectile dysfunction in the replication stage. (A) The forest diagram of MR study in the replication stage. (B–D) Mendelian randomization study of the effects of copper (B), vitamin D (C) and vitamin C (D) on erectile dysfunction in the replication stage. (E–G) Leave‐one‐out sensitivity analysis for this Mendelian randomization study of the effects of copper (E), vitamin D (F), and vitamin C (G) on erectile dysfunction in the replication stage.

In the replication stage, we identified copper (OR = 1.1151, 95% CI 1.0011–1.2420, *p* = 0.0476) and vitamin D (OR = 1.5607, 95% CI 1.0261–2.3738, *p* = 0.0375) as potential causal factors associated with ED using the IVW method (Figure 3A–C, Table S3). Conversely, circulating vitamin C did not exhibit any significant association with ED (OR = 0.8865, 95% CI 0.4794–1.6394, *p* = 0.7009, Figure 3A,D). Furthermore, no evidence of horizontal pleiotropy was detected through MR‐Egger, MR‐Egger regression, and MR‐PRESSO analyses (Figure S3, Table S3). The IVs employed for circulating vitamin D, copper, and other micronutrients in this stage of MR are detailed in Table 2 and Table S4. A leave‐one‐out sensitivity analysis demonstrated that the deletion of any single SNP related to copper and vitamin D did not significantly alter the outcomes (Table 3, Figure 3E–G, Figure S4), thereby affirming the robustness of the findings. Cochran's *Q* test was employed to evaluate the heterogeneity among the selected SNPs, and the IVW analysis showed no statistically significant heterogeneity (*p* > 0.05, Table S3). Based on these synthesized results, we tentatively hypothesize that circulating copper levels may serve as risk factors for ED.

### 3.2 The Effect of Micronutrients on ED by Pooled Analysis of Two GWAS‐Based Datasets

**FIGURE 4:**
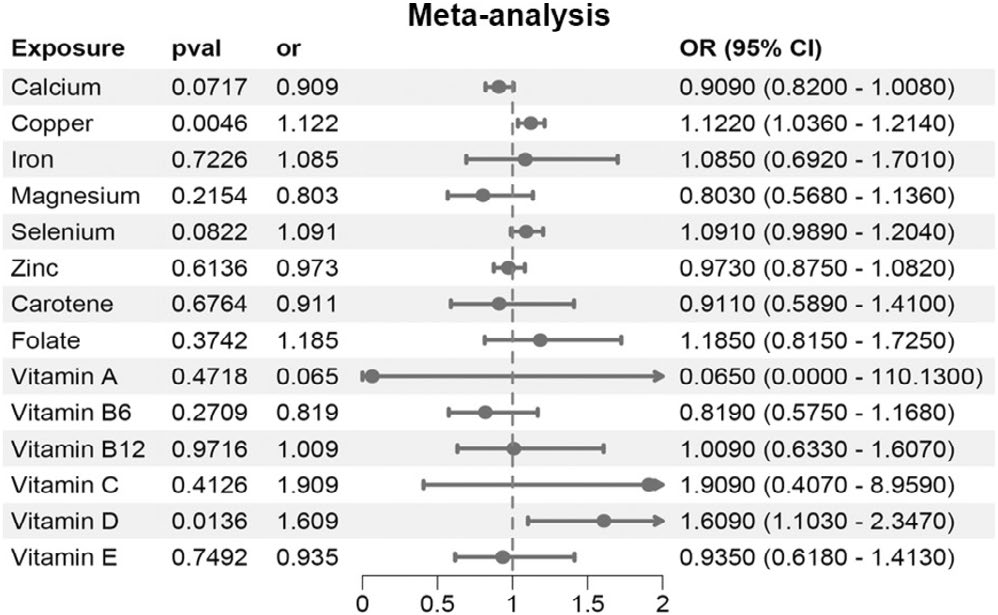
The causal association between micronutrition and erectile dysfunction by pooled analysis of two GWAS‐based datasets.

Furthermore, we conducted a pooled analysis of two GWAS‐based datasets using the aforementioned data to enhance the credibility of our study's findings. The results corroborated those observed during the replication phase, indicating a potential causal association between copper (OR = 1.122, 95% CI 1.0360–1.2140, *p* = 0.0046) and vitamin D (OR = 1.6090, 95% CI 1.1030–2.3470, *p* = 0.0136) with ED (Figure 4, Table S5). This outcome validates our hypothesis that circulating copper levels may serve as risk factors for ED.

### 3.3 Reverse Mendelian Randomization Analysis

We also examined the potential influence of the disease state of ED on the concentrations of copper, vitamin D, and other micronutrients. We applied the same MR methodology as previously described. In order to investigate the causal effects of eating disorders (ED) on micronutrient levels, we selected 139 SNPs in the discovery stage and 86 SNPs in the replication stage. All selected instruments demonstrated *F*‐statistic values exceeding 10. Our analyses, employing IVW, MR‐Egger, and weighted median regression methods, revealed no evidence of an effect of ED on vitamin D, copper, or other micronutrients in both the discovery and replication stages (Figure S5a–d, Tables S6 and S7). The results of the MR‐PRESSO test were consistent with those obtained using the aforementioned methods (Tables S6 and S7). We conducted an MR‐Egger regression to evaluate the presence of pleiotropy, and the findings indicated that horizontal pleiotropy was unlikely to affect the causal relationship between ED and micronutrients (Figures S6 and S8, Tables S6 and S7). The IVs utilized in the discovery and replication stages for circulating vitamin D, copper, and other micronutrients in the MR analysis are detailed in Tables S8 and S9. Additionally, the leave‐one‐out analysis demonstrated that no single SNP drove the MR estimates, thereby confirming the robustness of the results (Table 3, Figures S5e,f, S7, and S9). The findings of the pooled analysis suggest that ED does not exert a significant influence on the concentrations of vitamin D, copper, or other micronutrients (Figure S5g, Table S10).

## 4 Discussion

We examined the association between micronutrient concentrations and the risk of ED by enhancing causal inference in observational epidemiology through the use of MR. This study presents strong evidence of a causal relationship between genetically predicted higher copper levels and ED through the implementation of bidirectional MR, further validated by pooled analysis and sensitivity assessments. By employing this systematic and integrative methodology, the research offers a comprehensive evaluation of causality, thereby strengthening the reliability of its conclusions. This study highlights the critical role of maintaining nutritional balance, with a specific focus on copper intake, as an essential factor for sustaining sexual health. We emphasize the necessity of addressing this balance within nutritional management and clinical practice to prevent potential health risks associated with improper copper intake.

Erectile function is predominantly regulated by the endothelial cells within the corpus cavernosum (Tostes et al. 2008). Within these endothelial cells, endothelial nitric oxide synthase (eNOS) facilitates the conversion of L‐arginine to L‐citrulline, thereby promoting the release of nitric oxide (NO). The released NO activates guanylate cyclase (GC), which subsequently catalyzes the conversion of guanosine triphosphate (GTP) into cyclic guanosine monophosphate (cGMP) (Tostes et al. 2008). This biochemical cascade culminates in the relaxation of smooth muscle tissue within the corpus cavernosum, ultimately initiating penile erection.

Endothelial dysfunction and the induction of oxidative stress are principal factors contributing to ED, often characterized by the impaired capacity of endothelial cells to generate adequate levels of bioactive NO (Kondoh et al. 2008). The equilibrium of micronutrients plays a vital role in sustaining overall health, especially in the realm of sexual health, where they modulate vascular function and support endothelial cell activity through diverse physiological mechanisms. Copper is a crucial micronutrient that significantly contributes to the maintenance of endothelial function and the promotion of NO production (Crafa et al. 2020; Wei et al. 2019), which can be detected from human blood by inductively coupled mass spectrometry performance (Kojo et al. 2025). Whereas, genetic variants associated with circulating micronutrients levels were performed in an MR study (Fan et al. 2023; Miao et al. 2023).

Copper, as an indispensable mineral, is integral to numerous physiological processes, especially in the context of antioxidant defense and cardiovascular health. It is a vital constituent of several enzymes, including copper/zinc superoxide dismutase (SOD) and ceruloplasmin (CP), which facilitate reactions that neutralize free radicals and sustain cellular function (Al‐Bayati et al. 2015). However, maintaining copper homeostasis is of paramount importance, given that excessive copper intake can have adverse effects (Feng et al. 2023). Research has demonstrated that an excess of copper can induce oxidative stress, leading to the overproduction of reactive oxygen species (ROS) (Gaetke and Chow 2003; Shimada et al. 2018). These ROS interact with NO to form peroxynitrite (ONOO−), thereby diminishing NO bioavailability and resulting in endothelial dysfunction, a condition that contributes to the pathogenesis of ED (Agarwal et al. 2006).

Further investigations have elucidated that surplus copper ions are capable of being conveyed by CP to the smooth muscle cells and endothelial cells within the penile corpus cavernosum (Sullivan et al. 2005). In these locations, they inhibit the activity of nitric oxide synthase (NOS) (Perry et al. 2000; Perry and Marletta 1998) and facilitate the oxidation of NO into nitrosonium ions (NO^+^), thereby further reducing NO bioavailability and ultimately compromising erectile function (Bielli and Calabrese 2002; Torres and Wilson 1999). For instance, Animal experiments have demonstrated that diabetic rats with ED exhibited higher levels of CP protein in penile tissues, leading to enhanced Cu^2+^ transfer to cavernosal smooth muscle cells and the endothelium (Sullivan et al. 2005). Furthermore, two cross‐sectional surveys have identified a markedly elevated risk of ED among individuals with chronic exposure to high copper levels (Musa et al. 2023), such as copper miners, thereby reinforcing the association between excessive copper and ED. This dual action highlights a mechanism by which copper ions, modulated by CP, may impact the intricate balance of NO, contributing to the dysregulation of penile erection.

Moreover, serum testosterone levels and testicular function could be the factors of ED (Jannini et al. 2014). Prior reports have indicated negative effects of circulating copper levels on testosterone secretion (Xiao et al. 2022), and the surplus of essential neurometals Copper (Kawahara et al. 2023; Tanaka et al. 2025) can aggravate mood disorders (Baj et al. 2023), and even contribute to diminished testicular function (Rotter et al. 2020). Correspondingly, ED has occurred in these patients (Tanaka et al. 2025).

Prior reports have indicated positive effects of circulating zinc levels on ED (Kawahara et al. 2023; Tanaka et al. 2025). However, while observational studies suggest an inverse relationship between zinc and copper in ED pathogenesis, our MR analysis found no genetic evidence linking zinc to ED risk. This discrepancy highlights the advantage of MR in disentangling direct causal effects from environmental confounding. The copper‐specific association persisted even after rigorous adjustment for correlated micronutrients, underscoring its unique role in endothelial dysfunction via oxidative stress and nitric oxide depletion. Future studies may explore whether copper‐zinc ratios, rather than absolute levels, modulate ED risk.

Previous studies have shown that vitamin D deficiency may be related to ED (Crafa et al. 2020; Wei et al. 2019) and that vitamin D supplementation has some therapeutic effect (Canguven et al. 2017; Tirabassi et al. 2018). Vitamin D is a solid alcohol derivative that is mainly produced by the skin in response to sunlight. Vitamin D augments NOS activity and elevates NO production, thereby enhancing endothelial function and facilitating vasodilation, which are critical for maintaining normal erectile function. In instances of vitamin D deficiency, NO production is diminished, resulting in endothelial dysfunction and subsequent ED. Consequently, vitamin D supplementation has demonstrated potential therapeutic efficacy in mitigating symptoms of ED.

Nanomaterials have been extensively utilized in therapeutic and diagnostic domains, notably in targeted treatments for ED (Cheng et al. 2022; Han et al. 2010; Kim et al. 2015). Copper, as a prevalent metallic nanomaterial, has demonstrated considerable efficacy in cancer immunotherapy and antibacterial applications (Lu et al. 2024). Nonetheless, our investigation employing MR analysis indicates that elevated copper levels may constitute a risk factor for ED, underscoring the necessity for meticulous regulation of copper intake and application. Future research should focus on identifying more suitable nanomaterials to develop effective and safe targeted therapies for ED.

While our study provides novel insights into the causal role of copper in ED, several limitations must be acknowledged: First, the genetic instruments and outcome data were derived exclusively from European‐ancestry populations. Generalizability to other ethnic groups remains uncertain, particularly given variations in micronutrient metabolism influenced by genetic ancestry, dietary patterns, and environmental exposures. Second, while we selected IVs at a suggestive threshold of *p* < 5 × 10^−6^, this permissive approach risks including weak or pleiotropic variants. However, the *F*‐statistics for all IVs exceeded 10, substantially mitigating weak instrument bias. Third, the comprehensive analysis of certain micronutrients was constrained by the absence of stratified data categorization regarding sex, age, dietary patterns, micronutrient supplementation status, or comorbidities. Future large‐scale clinical investigations should prioritize validating these observational outcomes while elucidating the pathophysiological mechanisms involved.

In summary, this study elucidates the mechanisms by which copper impacts erectile function and establishes a causal relationship between excessive copper levels and ED. These findings offer a theoretical foundation and propose new directions for future research aimed at developing comprehensive prevention and treatment strategies, including targeted therapies for ED through maintaining the nutrient balance.

## 5 Conclusion

Although adequate micronutrient intake is essential for overall health and erectile function, excessive copper levels may elevate the risk of ED. The research highlights that nutritional balance, particularly in terms of copper intake, should be regarded as a key factor in maintaining sexual health and should be considered in nutritional management and clinical practice to prevent potential risks and mitigate the harmful effects associated with improper intake.

## Author Contributions

**Zilong Wang:** conceptualization (equal), data curation (equal), formal analysis (equal), investigation (equal), methodology (equal), writing – review and editing (equal). **Zhen Xu:** conceptualization (equal), data curation (equal), formal analysis (equal), project administration (equal), writing – review and editing (equal). **Meilu Li:** conceptualization (equal), data curation (equal), formal analysis (equal), investigation (equal), writing – review and editing (equal). **Zhenghao Li:** investigation (equal), methodology (equal), resources (equal), software (equal). **Dandan Li:** project administration (equal), supervision (equal), validation (equal), visualization (equal), writing – original draft (equal). **Changze Song:** funding acquisition (equal), project administration (equal), supervision (equal), validation (equal), visualization (equal), writing – original draft (equal). **Xiaobin Wang:** conceptualization (equal), funding acquisition (equal), project administration (equal), supervision (equal), validation (equal), visualization (equal), writing – original draft (equal).

## Ethics Statement

The authors have nothing to report.

## Conflicts of Interest

The authors declare no conflicts of interest.

## Supporting information


**FIGURE S1.** Mendelian randomization study of the effects of calcium (a), iron (b), magnesium (c), selenium (d), zinc (e), carotene (f), folate (g), vitamin A (h), vitamin B6 (i), vitamin B12 (j), vitamin E (k) on erectile dysfunction in the discovery stage.
**FIGURE S2.** Leave‐one‐out sensitivity analysis for this Mendelian randomization study of the effects of calcium (a), iron (b), magnesium (c), selenium (d), zinc (e), carotene (f), folate (g), vitamin A (h), vitamin B6 (i), vitamin B12 (j), vitamin E (k) on erectile dysfunction in the discovery stage.
**FIGURE S3.** Mendelian randomization study of the effects of calcium (a), iron (b), magnesium (c), selenium (d), zinc (e), carotene (f), folate (g), vitamin A (h), vitamin B6 (i), vitamin B12 (j), vitamin E (k) on erectile dysfunction in the replication stage.
**FIGURE S4.** Leave‐one‐out sensitivity analysis for this Mendelian randomization study of the effects of calcium (a), iron (b), magnesium (c), selenium (d), zinc (e), carotene (f), folate (g), vitamin A (h), vitamin B6 (i), vitamin B12 (j), vitamin E (k) on erectile dysfunction in the replication stage.
**FIGURE S5.** Reverse Mendelian randomization study of the effects of erectile dysfunction in the discovery stage (a, b) and replication stage (c, d). Leave‐one‐out sensitivity analysis for this Mendelian randomization study of the effects of erectile dysfunction on circulating cop‐per levels (e) in the discovery stage and circulating vitamin D levels (f) on in the replication stage. (g) The causal association between erectile dysfunction and micronutrition by pooled analysis of two GWAS‐based datasets.
**FIGURE S6.** Mendelian randomization study of the effects of erectile dysfunction on calcium (a), iron (b), magnesium (c), selenium (d), zinc (e), carotene (f), folate (g), vitamin A (h), vitamin B6 (i), vitamin B12 (j), vitamin C (k) and vitamin E (l) in the discovery stage.
**FIGURE S7.** Leave‐one‐out sensitivity analysis for this Mendelian randomization study of the effects of erectile dysfunction on calcium (a), iron (b), magnesium (c), selenium (d), zinc (e), carotene (f), folate (g), vitamin A (h), vitamin B6 (i), vitamin B12 (j), vitamin C (k), and vitamin E (l) in the discovery stage.
**FIGURE S8.** Mendelian randomization study of the effects of erectile dysfunction on iron (a), magnesium (b), carotene (c), folate (d), vitamin A (e), vitamin B6 (f), vitamin B12 (g), vitamin C (h), vitamin E (i) in the replication stage.
**FIGURE S9.** Leave‐one‐out sensitivity analysis for this Mendelian randomization study of the effects of erectile dysfunction on iron (a), magnesium (b), carotene (c), folate (d), vitamin A (e), vitamin B6 (f), vitamin B12 (g), vitamin C (h), vitamin E (i) in the replication stage.

**TABLE S1.** Mendelian randomization estimation for micronutrients on the risk of erectile dysfunction in the discovery stage.
**TABLE S2.** The instrumental variables for the associations of the micronutrients‐associated SNPs with these exposures and erectile dysfunction in the discovery stage.
**TABLE S3.** Mendelian Randomization estimation for micronutrients on the risk of erectile dysfunction in the replication stage.
**TABLE S4.** The instrumental variables for the associations of the micronutrients‐associated SNPs with these exposures and erectile dysfunction in the replication stage.
**TABLE S5.** Summary of pooled analysis of two GWAS‐based datasets on the causal association between micronutrients and erectile dysfunction.
**TABLE S6.** Reverse Mendelian randomization estimation for erectile dysfunction on the level of micronutrients in the discovery stage.
**TABLE S7.** Reverse Mendelian randomization estimation for erectile dysfunction on the level of micronutrients in the replication stage.
**TABLE S8.** Summary statistics for the associations of the erectile dysfunction‐associated SNPs with these exposures and micronutrients in the discovery stage.
**TABLE S9.** Summary statistics for the associations of the erectile dysfunction‐associated SNPs with these exposures and micronutrients in the replication stage.
**TABLE S10.** Summary of pooled analysis of two GWAS‐based datasets on the causal association between erectile dysfunction and micronutrients.

## Data Availability

The datasets analyzed for this study can be found in the FinnGen Biobank (dataset: finn‐b‐ERECTILE_DYSFUNCTION) (Kurki et al. 2023) as the discovery stage and GWAS conducted by Bovijn J. et al. (dataset: ebi‐a‐GCST006956) as the replication stage (Bovijn et al. 2019). All GWAS summary statistics utilized in this work are publicly available through the IEU Open GWAS database (https://gwas.mrcieu.ac.uk/).
