# Supplementary material for: Unraveling the Causal Association Between Circulating Copper Levels and Erectile Dysfunction: A Comprehensive Analysis via Bidirectional Two Sample Mendelian Randomization Study
Source: Food Sci Nutr. 2025 May 26;13(6):e70247. doi: 10.1002/fsn3.70247 (PMC12121513; doi:10.1002/fsn3.70247)
Supplement: Supplementary file 1 — FIGURE S1. Mendelian randomization study of the effects of calcium (a), iron (b), magnesium (c), selenium (d), zinc (e), carotene (f), folate (g), vitamin A (h), vitamin B6 (i), vitamin B12 (j), vitamin E (k) on erectile dysfunction in the discovery stage. FIGURE S2. Leave‐one‐out sensitivity analysis for this Mendelian randomization study of the effects of calcium (a), iron (b), magnesium (c), selenium (d), zinc (e), carotene (f), folate (g), vitamin A (h), vitamin B6 (i), vitamin B12 (j), vitamin E (k) on erectile dysfunction in the discovery stage. FIGURE S3. Mendelian randomization study of the effects of calcium (a), iron (b), magnesium (c), selenium (d), zinc (e), carotene (f), folate (g), vitamin A (h), vitamin B6 (i), vitamin B12 (j), vitamin E (k) on erectile dysfunction in the replication stage. FIGURE S4. Leave‐one‐out sensitivity analysis for this Mendelian randomization study of the effects of calcium (a), iron (b), magnesium (c), selenium (d), zinc (e), carotene (f), folate (g), vitamin A (h), vitamin B6 (i), vitamin B12 (j), vitamin E (k) on erectile dysfunction in the replication stage. FIGURE S5. Reverse Mendelian randomization study of the effects of erectile dysfunction in the discovery stage (a, b) and replication stage (c, d). Leave‐one‐out sensitivity analysis for this Mendelian randomization study of the effects of erectile dysfunction on circulating cop‐per levels (e) in the discovery stage and circulating vitamin D levels (f) on in the replication stage. (g) The causal association between erectile dysfunction and micronutrition by pooled analysis of two GWAS‐based datasets. FIGURE S6. Mendelian randomization study of the effects of erectile dysfunction on calcium (a), iron (b), magnesium (c), selenium (d), zinc (e), carotene (f), folate (g), vitamin A (h), vitamin B6 (i), vitamin B12 (j), vitamin C (k) and vitamin E (l) in the discovery stage. FIGURE S7. Leave‐one‐out sensitivity analysis for this Mendelian randomization study of the effects [file FSN3-13-e70247-s002.docx]

Supplementary Material

## Supplementary Figures

**Supplementary Figure S1.** Mendelian randomization study of the effects of calcium (a), iron (b), magnesium (c), selenium (d), zinc (e), carotene (f), folate (g), vitamin A (h), vitamin B6 (i), vitamin B12 (j), vitamin E (k) on erectile dysfunction in the discovery stage.

**Supplementary Figure S2.** Leave-one-out sensitivity analysis for this Mendelian randomization study of the effects of calcium (a), iron (b), magnesium (c), selenium (d), zinc (e), carotene (f), folate (g), vitamin A (h), vitamin B6 (i), vitamin B12 (j), vitamin E (k) on erectile dysfunction in the discovery stage.

**Supplementary Figure S3.** Mendelian randomization study of the effects of calcium (a), iron (b), magnesium (c), selenium (d), zinc (e), carotene (f), folate (g), vitamin A (h), vitamin B6 (i), vitamin B12 (j), vitamin E (k) on erectile dysfunction in the replication stage.

**Supplementary Figure S4.** Leave-one-out sensitivity analysis for this Mendelian randomization study of the effects of calcium (a), iron (b), magnesium (c), selenium (d), zinc (e), carotene (f), folate (g), vitamin A (h), vitamin B6 (i), vitamin B12 (j), vitamin E (k) on erectile dysfunction in the replication stage.

**Supplementary figure S5.** Reverse Mendelian randomization study of the effects of erectile dysfunction in the discovery stage (a-b) and replication stage (c-d). Leave-one-out sensitivity analysis for this Mendelian randomization study of the effects of erectile dysfunction on circulating cop-per levels (e) in the discovery stage and circulating vitamin D levels (f) on in the replication stage. (g) The causal association between erectile dysfunction and micronutrition by pooled analysis of two GWAS-based datasets.

**Supplementary Figure S6.** Mendelian randomization study of the effects of erectile dysfunction on calcium (a), iron (b), magnesium (c), selenium (d), zinc (e), carotene (f), folate (g), vitamin A (h), vitamin B6 (i), vitamin B12 (j), vitamin C (k) and vitamin E (l) in the discovery stage.

**Supplementary Figure S7.** Leave-one-out sensitivity analysis for this Mendelian randomization study of the effects of erectile dysfunction on calcium (a), iron (b), magnesium (c), selenium (d), zinc (e), carotene (f), folate (g), vitamin A (h), vitamin B6 (i), vitamin B12 (j), vitamin C (k) and vitamin E (l) in the discovery stage.

**Supplementary Figure S8.** Mendelian randomization study of the effects of erectile dysfunction on iron (a), magnesium (b), carotene (c), folate (d), vitamin A (e), vitamin B6 (f), vitamin B12 (g), vitamin C (h), vitamin E (i) in the replication stage.

**Supplementary Figure S9.** Leave-one-out sensitivity analysis for this Mendelian randomization study of the effects of erectile dysfunction on iron (a), magnesium (b), carotene (c), folate (d), vitamin A (e), vitamin B6 (f), vitamin B12 (g), vitamin C (h), vitamin E (i) in the replication stage.


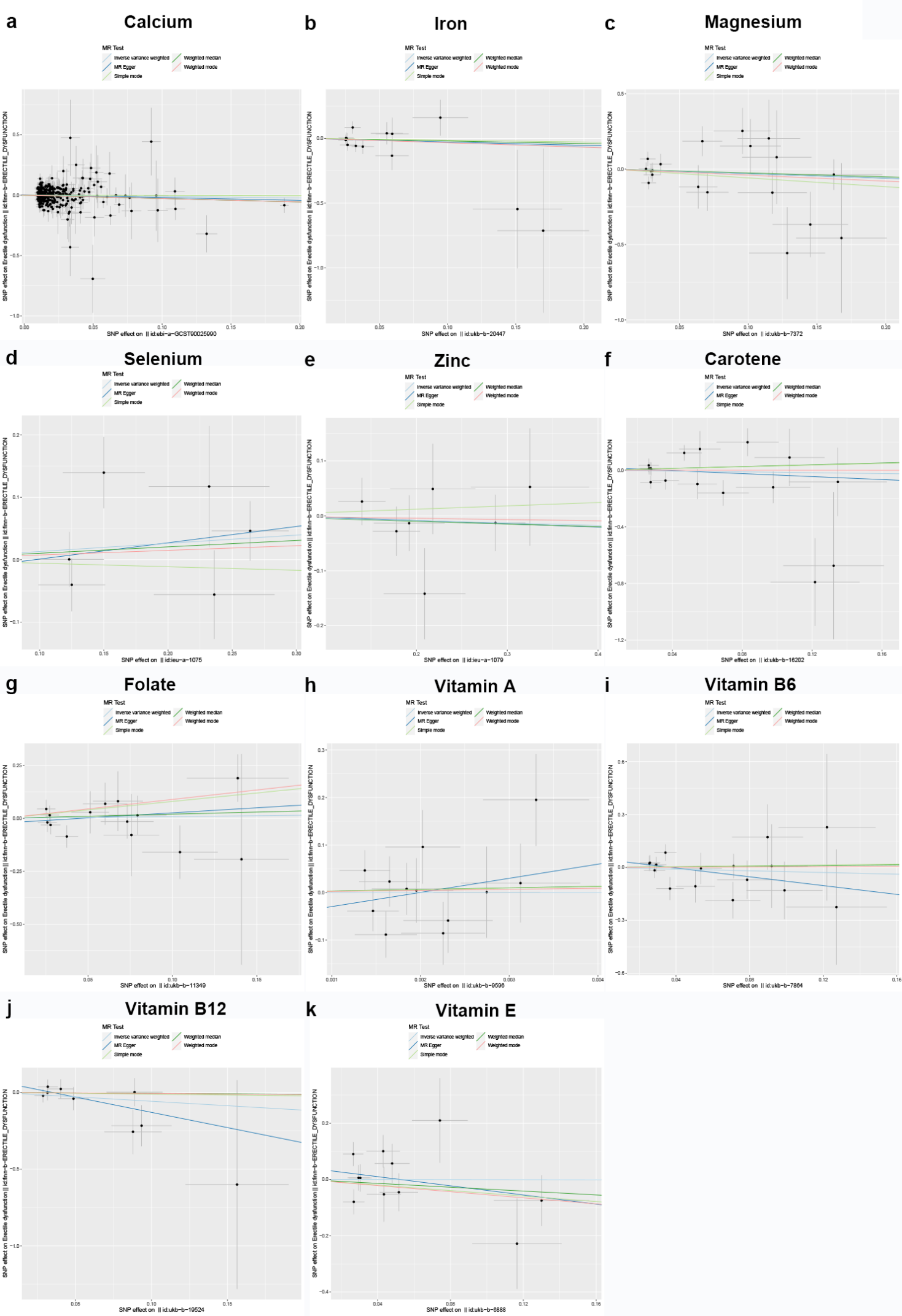


**Supplementary Figure S1** Mendelian randomization study of the effects of calcium (a), iron (b), magnesium (c), selenium (d), zinc (e), carotene (f), folate (g), vitamin A (h), vitamin B6 (i), vitamin B12 (j), vitamin E (k) on erectile dysfunction in the discovery stage.


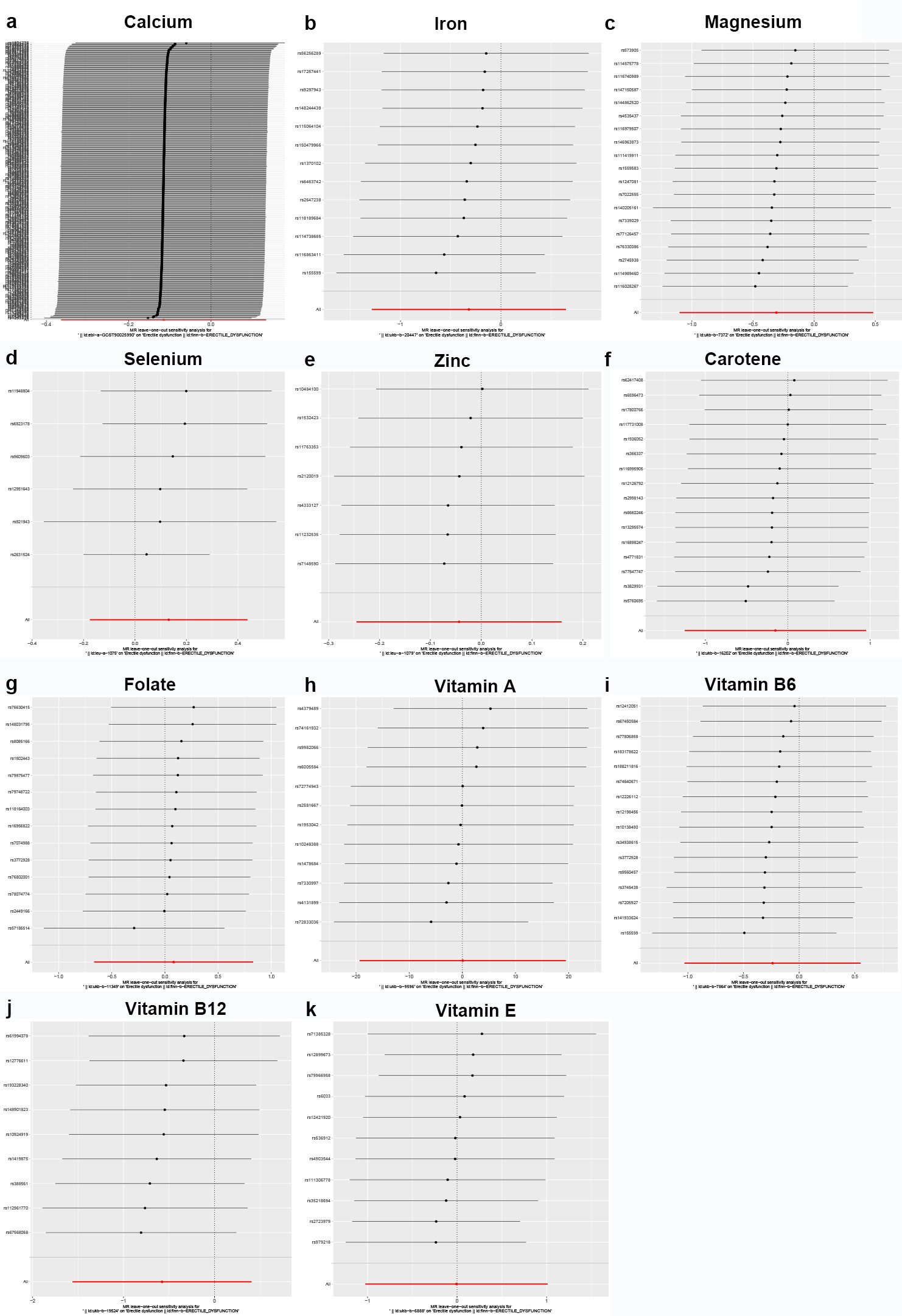


**Supplementary Figure S2** Leave-one-out sensitivity analysis for this Mendelian randomization study of the effects of calcium (a), iron (b), magnesium (c), selenium (d), zinc (e), carotene (f), folate (g), vitamin A (h), vitamin B6 (i), vitamin B12 (j), vitamin E (k) on erectile dysfunction in the discovery stage.


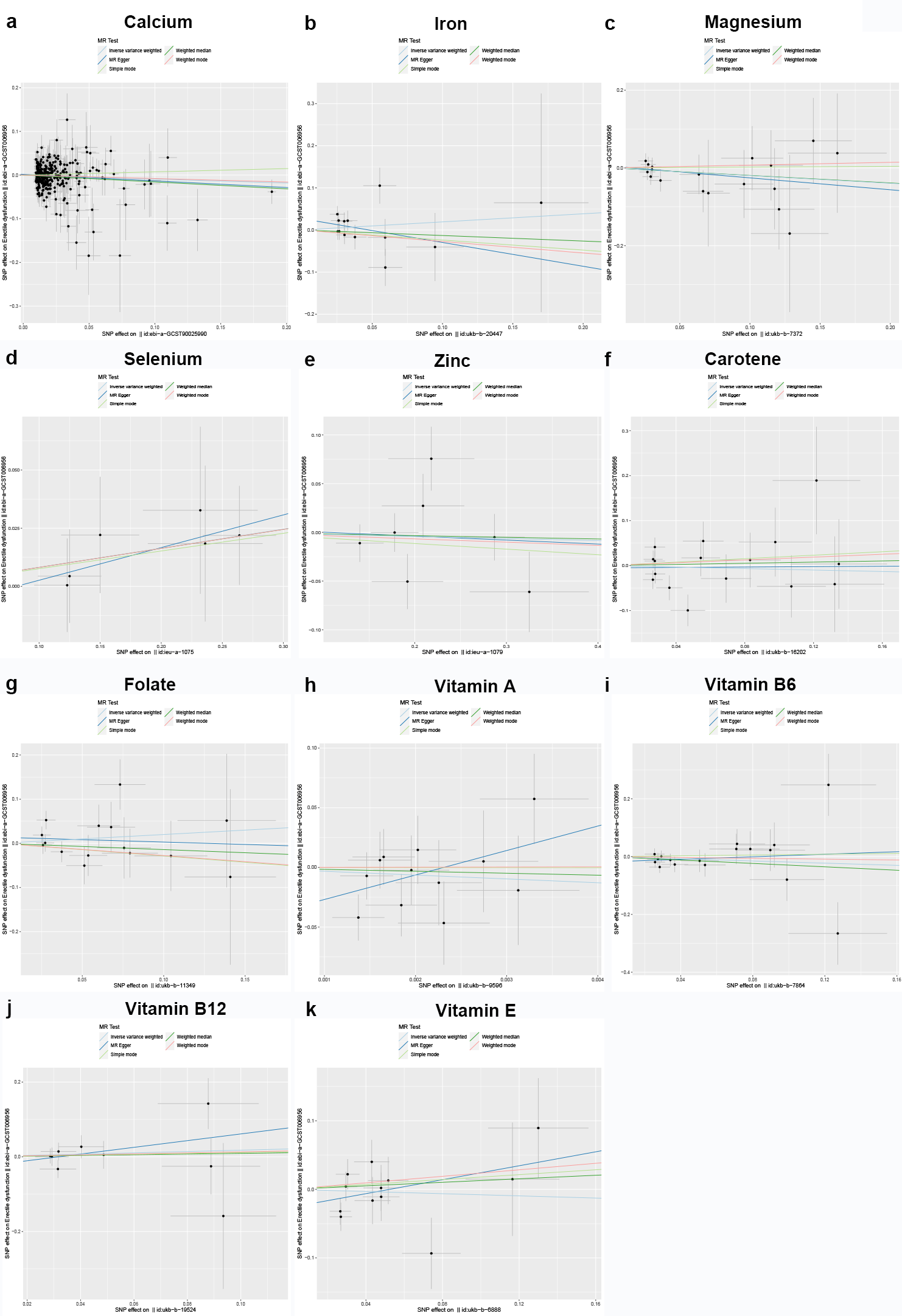


**Supplementary Figure S3** Mendelian randomization study of the effects of calcium (a), iron (b), magnesium (c), selenium (d), zinc (e), carotene (f), folate (g), vitamin A (h), vitamin B6 (i), vitamin B12 (j), vitamin E (k) on erectile dysfunction in the replication stage.


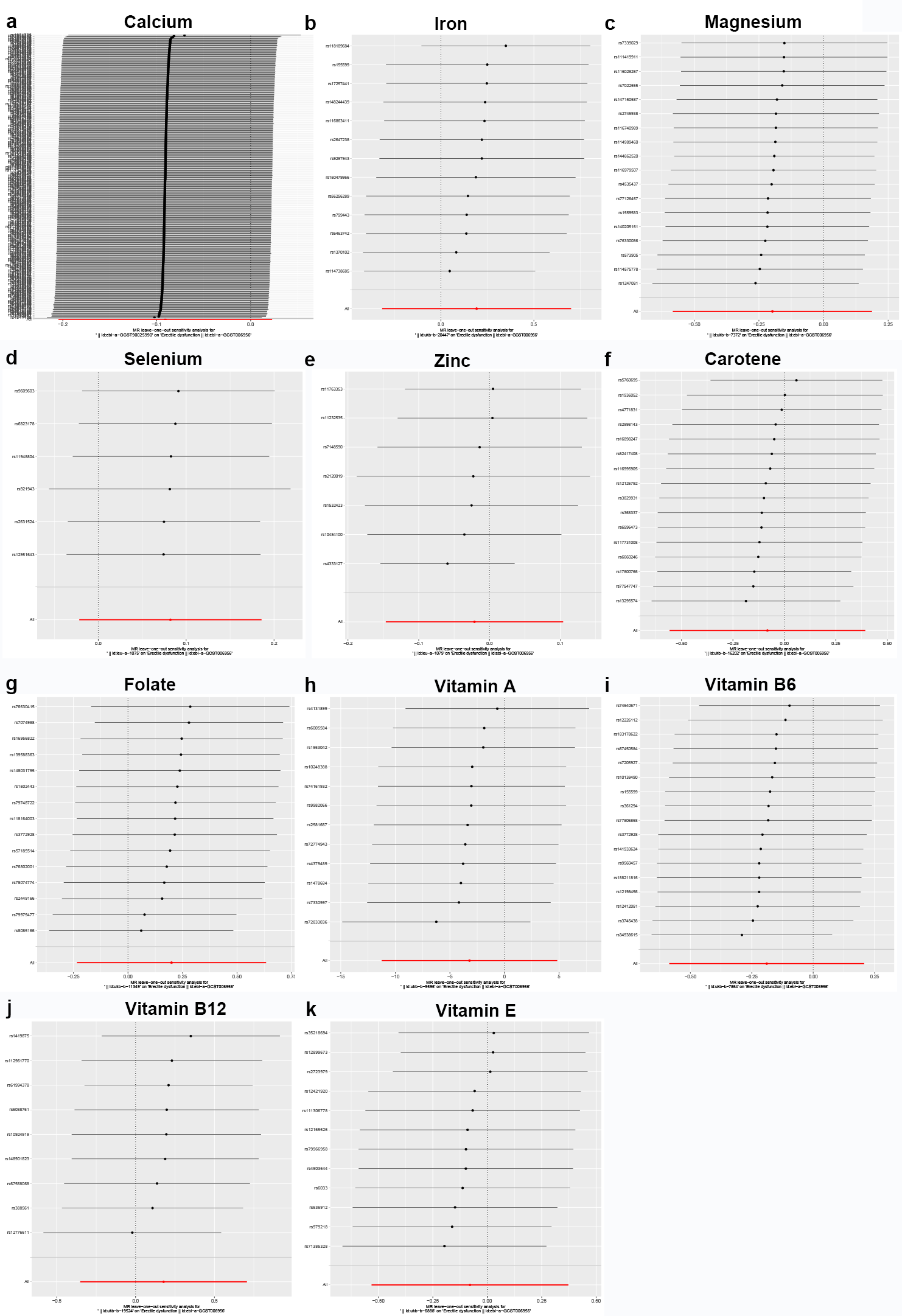


**Supplementary Figure S4** Leave-one-out sensitivity analysis for this Mendelian randomization study of the effects of calcium (a), iron (b), magnesium (c), selenium (d), zinc (e), carotene (f), folate (g), vitamin A (h), vitamin B6 (i), vitamin B12 (j), vitamin E (k) on erectile dysfunction in the replication stage.


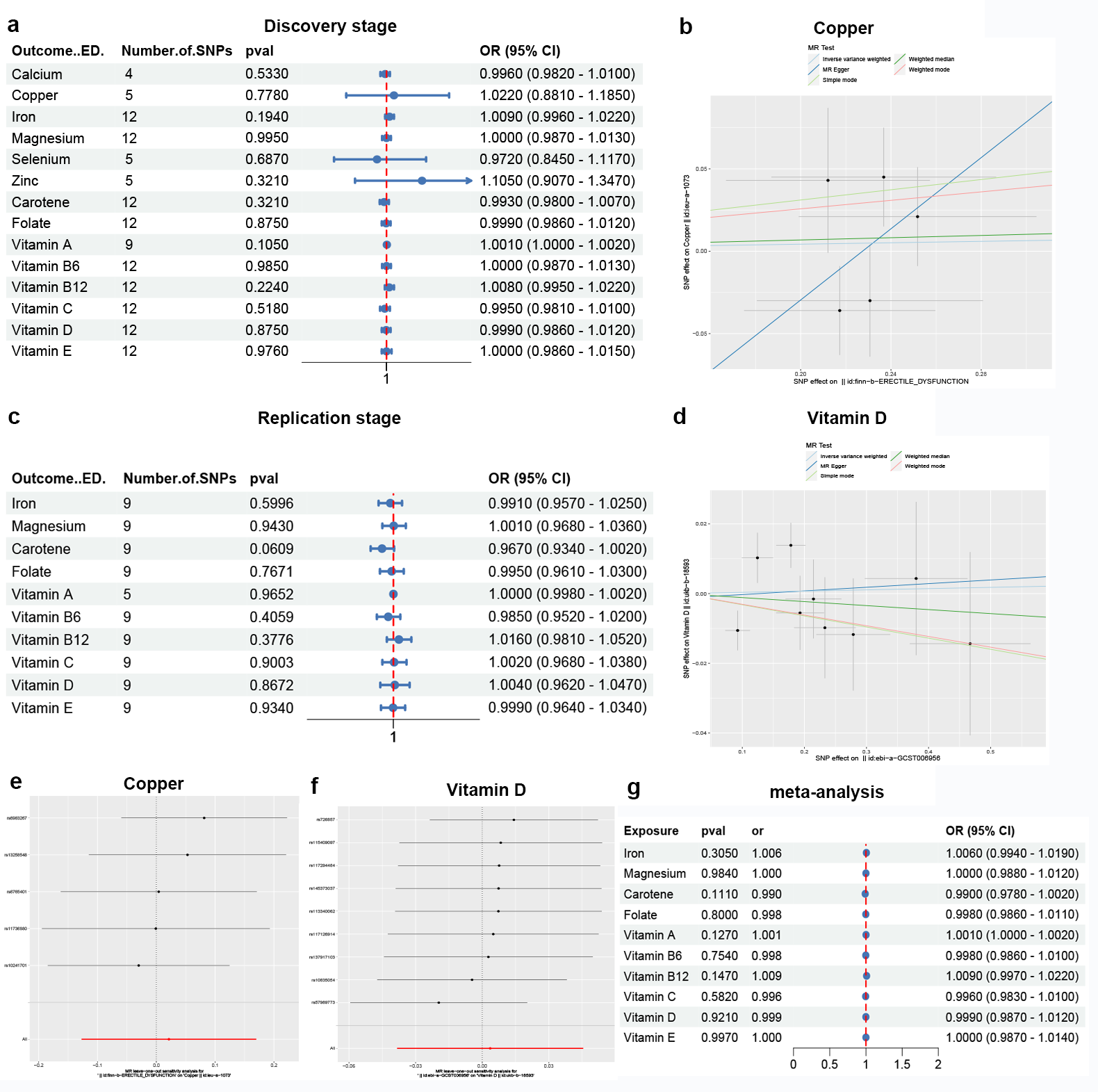


**Supplementary figure S5** Reverse Mendelian randomization study of the effects of erectile dysfunction in the discovery stage (a-b) and replication stage (c-d). Leave-one-out sensitivity analysis for this Mendelian randomization study of the effects of erectile dysfunction on circulating copper levels (e) in the discovery stage and circulating vitamin D levels (f) on in the replication stage. (g) The causal association between erectile dysfunction and micronutrition by pooled analysis of two GWAS-based datasets.


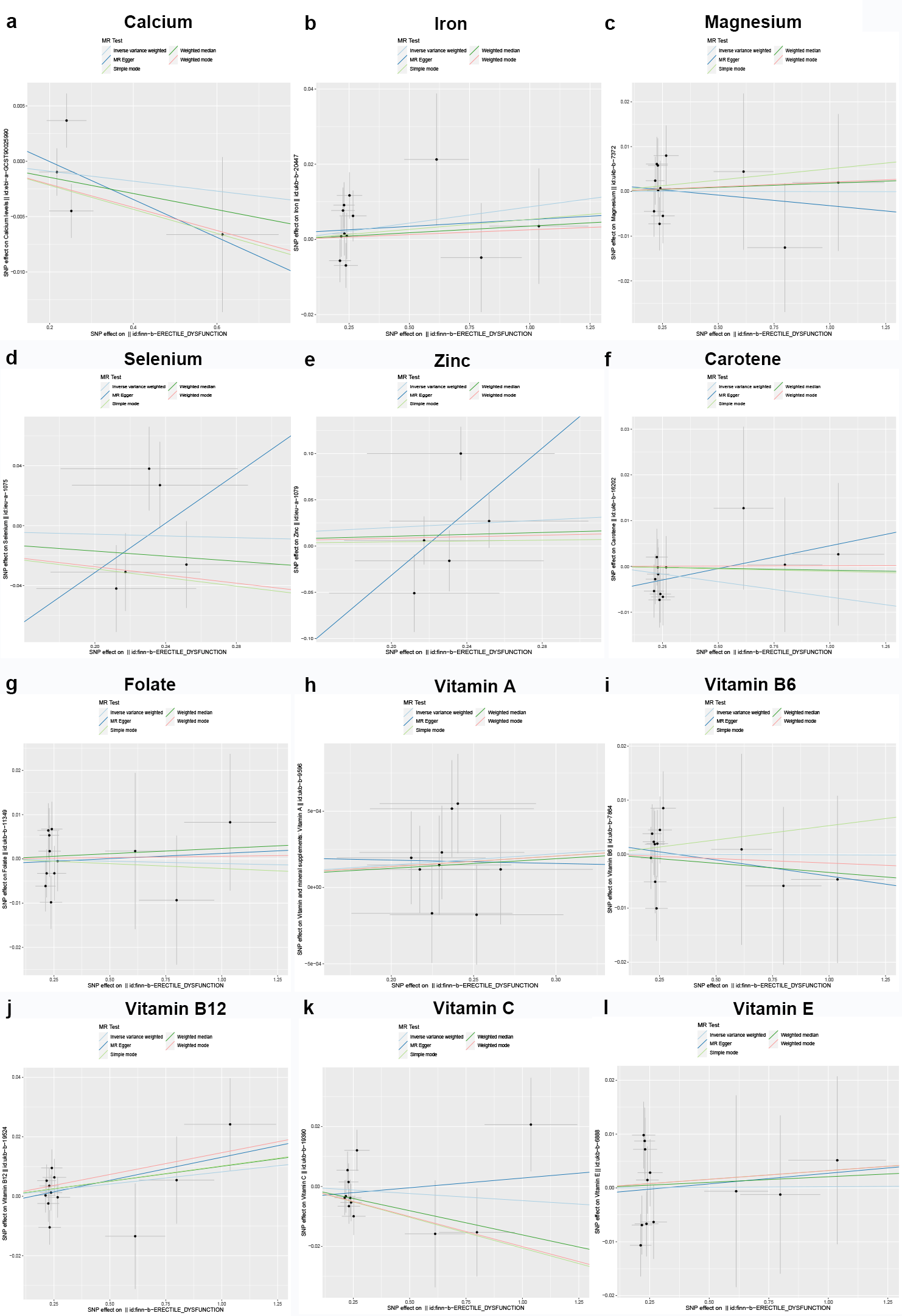


**Supplementary Figure S6** Mendelian randomization study of the effects of erectile dysfunction on calcium (a), iron (b), magnesium (c), selenium (d), zinc (e), carotene (f), folate (g), vitamin A (h), vitamin B6 (i), vitamin B12 (j), vitamin C (k) and vitamin E (l) in the discovery stage.


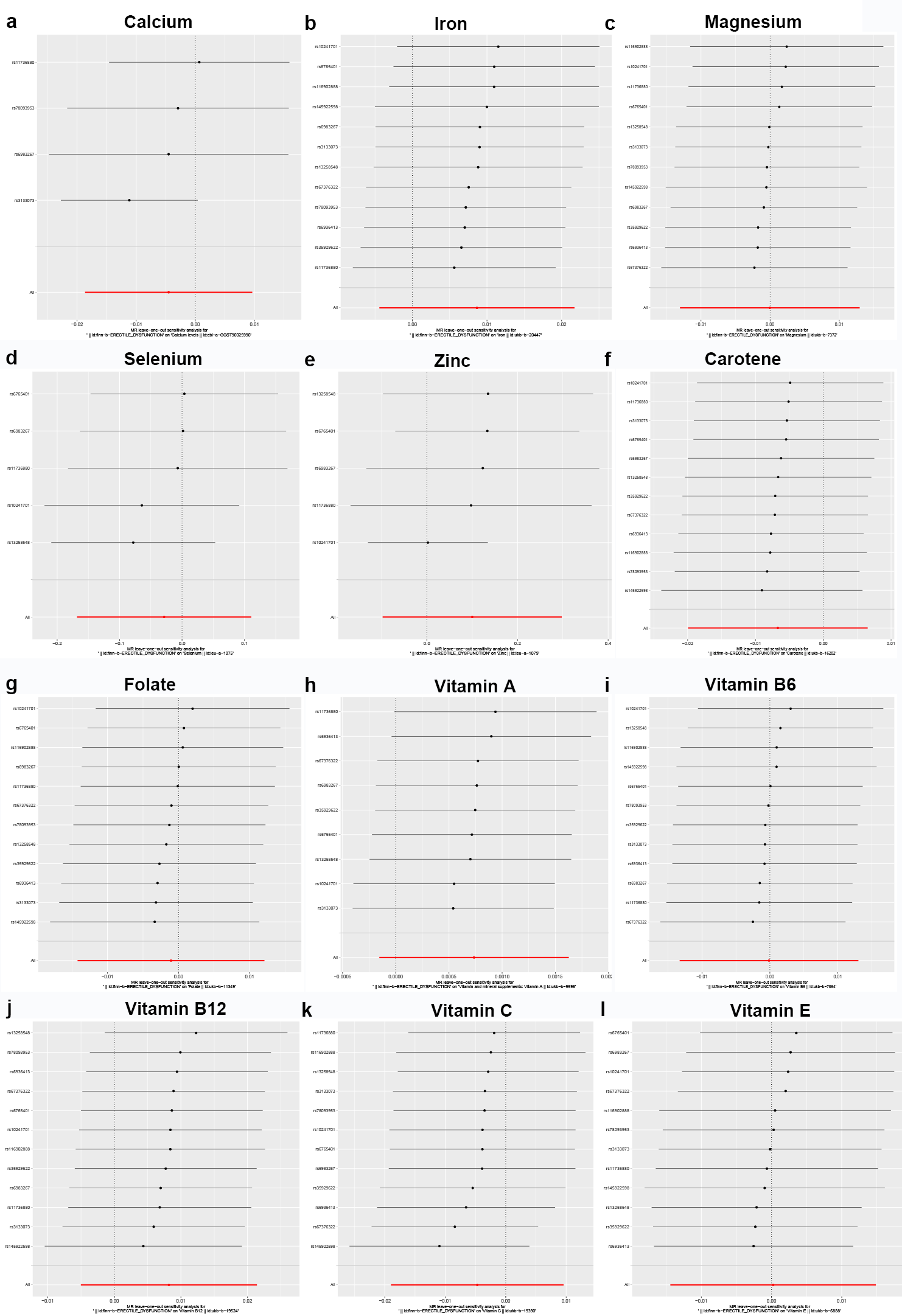


**Supplementary Figure S7** Leave-one-out sensitivity analysis for this Mendelian randomization study of the effects of erectile dysfunction on calcium (a), iron (b), magnesium (c), selenium (d), zinc (e), carotene (f), folate (g), vitamin A (h), vitamin B6 (i), vitamin B12 (j), vitamin C (k) and vitamin E (l) in the discovery stage.


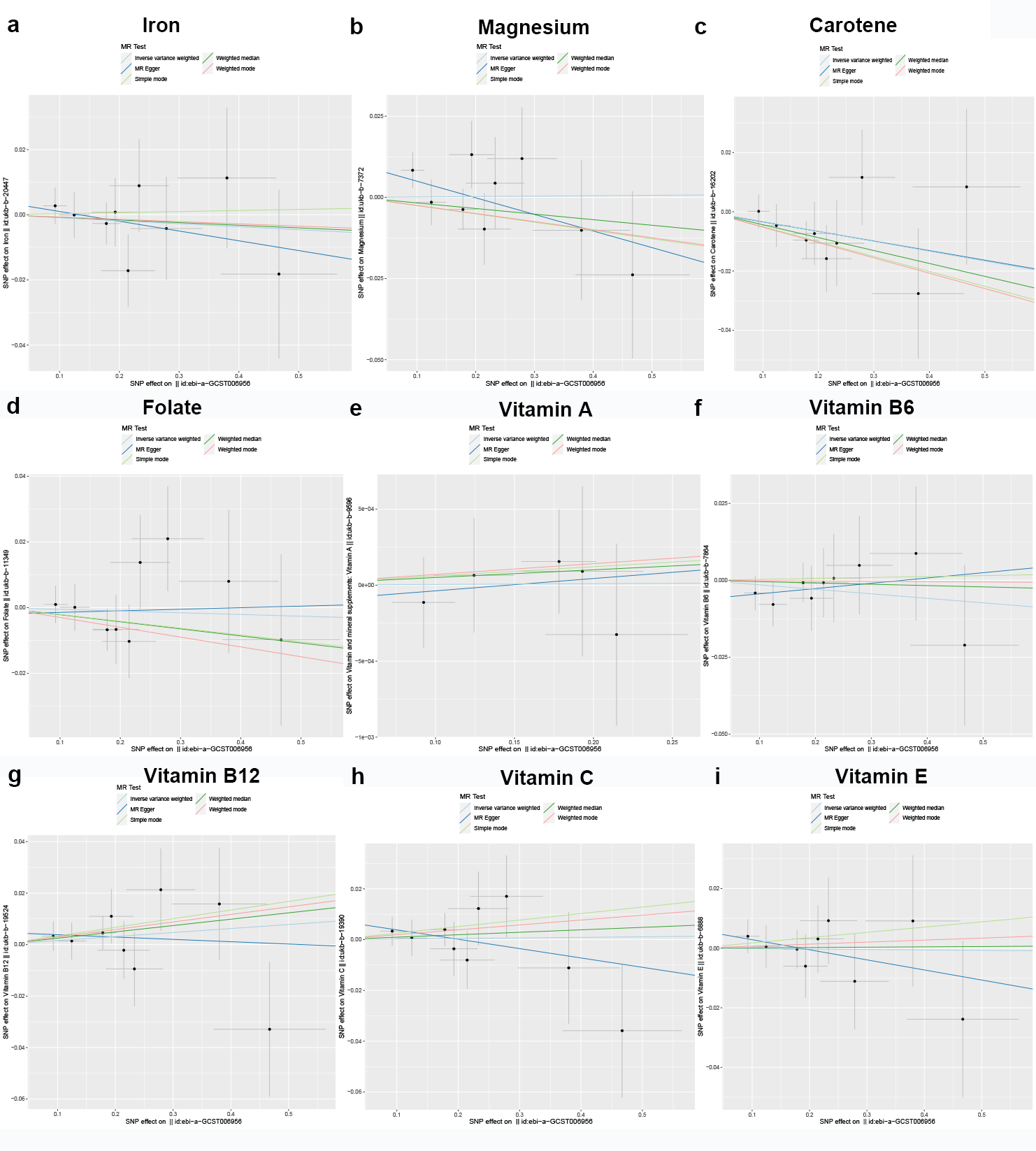


**Supplementary Figure S8** Mendelian randomization study of the effects of erectile dysfunction on iron (a), magnesium (b), carotene (c), folate (d), vitamin A (e), vitamin B6 (f), vitamin B12 (g), vitamin C (h), vitamin E (i) in the replication stage.


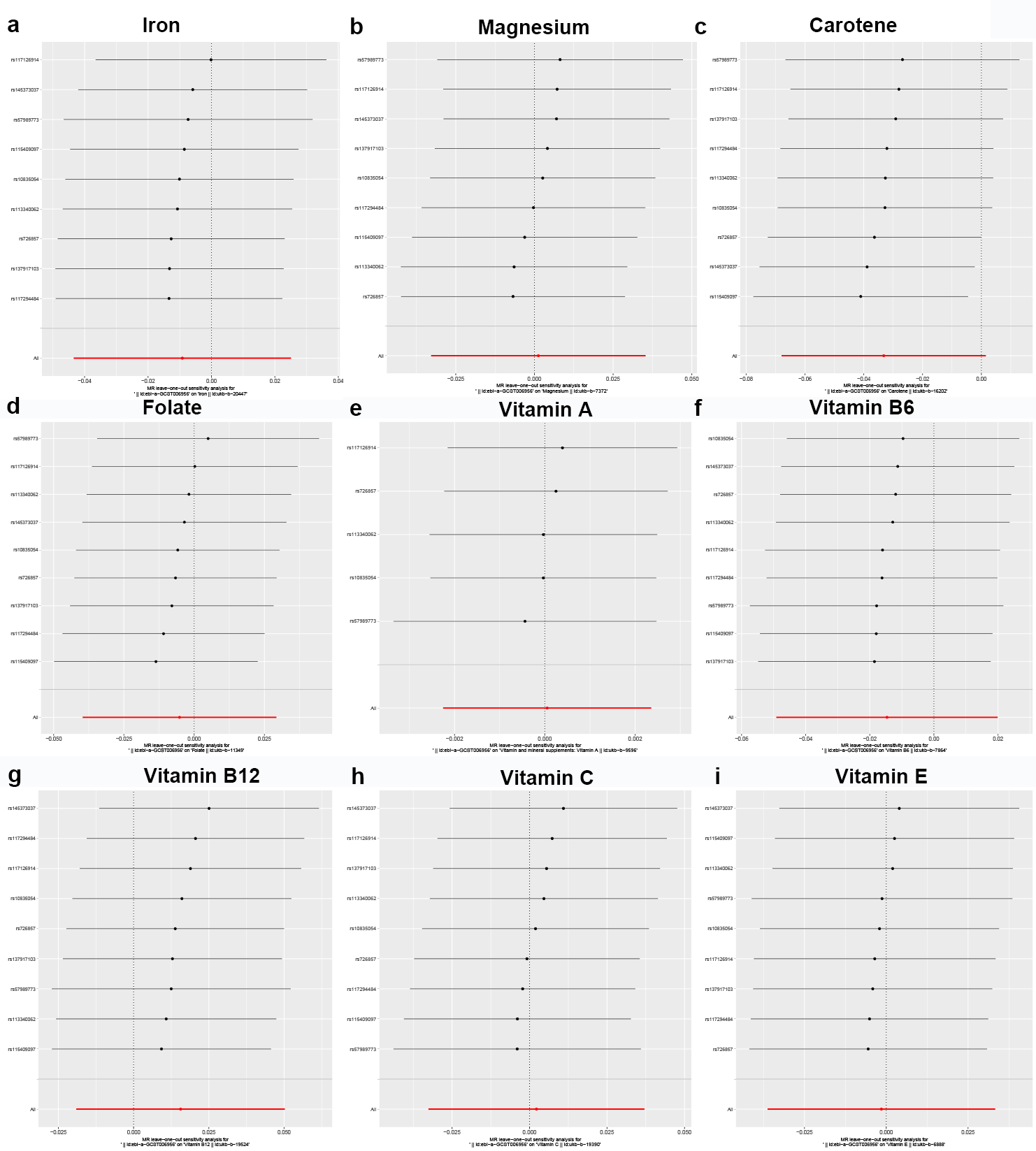


**Supplementary Figure S9** Leave-one-out sensitivity analysis for this Mendelian randomization study of the effects of erectile dysfunction on iron (a), magnesium (b), carotene (c), folate (d), vitamin A (e), vitamin B6 (f), vitamin B12 (g), vitamin C (h), vitamin E (i) in the replication stage.

## Supplementary Tables

**Supplementary Table S1.** Mendelian Randomization estimation for micronutrients on the risk of erectile dysfunction in the discovery stage.

**Supplementary Table S2.** The instrumental variables for the associations of the micronutrients-associated SNPs with these exposures and erectile dysfunction in the discovery stage.

**Supplementary Table S3.** Mendelian Randomization estimation for micronutrients on the risk of erectile dysfunction in the replication stage.

**Supplementary Table S4.** The instrumental variables for the associations of the micronutrients-associated SNPs with these exposures and erectile dysfunction in the replication stage.

**Supplementary Table S5.** Summary of pooled analysis of two GWAS-based datasets on the causal association between micronutrients and erectile dysfunction.

**Supplementary Table S6.** Reverse Mendelian Randomization estimation for erectile dysfunction on the level of micronutrients in the discovery stage.

**Supplementary Table S7.** Reverse Mendelian Randomization estimation for erectile dysfunction on the level of micronutrients in the replication stage.

**Supplementary Table S8.** Summary statistics for the associations of the erectile dysfunction-associated SNPs with these exposures and micronutrients in the discovery stage.

**Supplementary Table S9.** Summary statistics for the associations of the erectile dysfunction-associated SNPs with these exposures and micronutrients in the replication stage.

**Supplementary Table S10.** Summary of pooled analysis of two GWAS-based datasets on the causal association between erectile dysfunction and micronutrients.
